# Metabolic effects of trehalose in mice of the C57BL/6 strain with obesity induced by a high carbohydrate-fat diet

**DOI:** 10.18699/vjgb-25-88

**Published:** 2025-10

**Authors:** A.B. Pupyshev, N.M. Bazhan, A.Yu. Kazantseva, T.V. Yakovleva, V.M. Belichenko, N.V. Goncharova, T.A. Korolenko, M.A. Tikhonova

**Affiliations:** Scientific Research Institute of Neurosciences and Medicine, Novosibirsk, Russia; Institute of Cytology and Genetics of the Siberian Branch of the Russian Academy of Sciences, Novosibirsk, Russia; Institute of Cytology and Genetics of the Siberian Branch of the Russian Academy of Sciences, Novosibirsk, Russia; Institute of Cytology and Genetics of the Siberian Branch of the Russian Academy of Sciences, Novosibirsk, Russia; Scientific Research Institute of Neurosciences and Medicine, Novosibirsk, Russia; Scientific Research Institute of Neurosciences and Medicine, Novosibirsk, Russia; Scientific Research Institute of Neurosciences and Medicine, Novosibirsk, Russia; Scientific Research Institute of Neurosciences and Medicine, Novosibirsk, Russia

**Keywords:** C57BL/6 mice, carbohydrate-fat diet, cafeteria diet, obesity, trehalose, autophagy, qPCR, glucose, triglycerides, cholesterol, мыши С57BL/6, углеводножировая диета, диета кафетерия, ожирение, трегалоза, аутофагия, ПЦР, глюкоза, триглицериды, холестерин

## Abstract

The ability of trehalose to improve metabolic parameters in mice with experimental obesity has been shown to depend on the type of obesity model. In db/db mice, it reduced body weight, insulin, blood glucose, and cholesterol levels. In mice with obesity induced by high-fat dietary intake, it had no effect on body weight but reduced blood insulin levels with compensatory upregulation of insulin signaling gene expression. We studied the effect of trehalose on overweight and metabolic parameters in C57BL/6 inbred mice with obesity induced by a high carbohydrate-fat diet, the “cafeteria diet”. The cafeteria diet consisted of free access to water, standard chow, fatty foods (lard), and carbohydrates (biscuits) for 18 weeks. All mice were then randomly divided into four groups for four weeks of treatment: (1) water drinking, (2) drinking 3 % trehalose, (3) cafeteria diet and drinking water, (4) cafeteria diet and drinking 3 % trehalose. Alterations in body mass, food intake, fluid intake, dietary calories, blood biochemical parameters (glucose, triglyceride, cholesterol, HDL, ALT, creatinine levels), expression of carbohydrate metabolism (Slc2a2, Insr) and autophagy (Atg8, Becn1, Park2) genes in the liver were studied. The cafeteria diet obesity model was accompanied by some signs of metabolic syndrome as it induced an increase in body weight (by 25 %), calorie intake (by 25 %), blood levels of glucose (by 35 %), cholesterol (by 66 %), and triglycerides (by 23 %) in mice. Trehalose had little effect on control mice, causing a decrease in standard food intake and an increase in dietary caloric intake by the number of calories from trehalose itself. In obese mice, trehalose increased total caloric intake and biscuit consumption but had no substantial effect on body weight gain, blood metabolic parameters, or expression of liver genes regulating glucose transport (Slc2a2), insulin sensitivity (Insr), and autophagy processes (Atg8, Becn1, Park2). Since the cafeteria diet is the most adequate model of alimentary obesity development in humans, our results question the use of trehalose to correct the dietary type of obesity in humans.

## Abstract (ru)

Показано, что способность трегалозы улучшать метаболические показатели у животных с экспериментальным ожирением зависит от модели ожирения. У мышей линии db/db она снижает вес тела, уровни инсулина, глюкозы и холестерина в крови. У мышей с ожирением, вызванным потреблением высокожировой диеты, она не влияет на вес тела, но снижает уровень инсулина в крови, компенсаторно усиливая экспрессию генов инсулиновой сигнализации. Нами предпринято исследование действия трегалозы на вес и метаболические показатели у мышей линии C57BL/6 с избыточным весом, вызванным диетой с повышенным содержанием жиров и углеводов – «диетой кафетерия». Диета кафетерия включала свободный доступ на протяжении 18 недель к воде, стандартному корму, жирной пище (салу) и углеводам (сдобному печенью). Все мыши были рандомно разделены на четыре группы, содержавшиеся в разных условиях в течение 4 недель: 1) питье воды; 2) питье 3 % трегалозы; 3) диета кафетерия и питье воды; 4) диета кафетерия и питье 3 % трегалозы. Исследовали изменения массы тела, потребление корма, жидкости, пищевых калорий, биохимические показатели крови (уровень глюкозы, триглицеридов, холестерина, ЛПВП, АЛТ, креатинина), экспрессию генов углеводного обмена (Slc2a2, Insr) и аутофагии (Atg8, Becn1, Park2) в печени. Модель ожирения с помощью диеты кафетерия сопровождалась признаками метаболического синдрома, поскольку у этих мышей были повышены: масса тела (на 25 %), количество потребленных калорий (на 20 %), уровни в крови глюкозы (на 35 %), холестерина (на 66 %), триглицеридов (на 23 %). На контрольных мышей трегалоза действовала слабо, вызывая лишь снижение потребления стандартного корма и повышение потребления пищевых калорий на величину калорийности самой трегалозы. У мышей с ожирением трегалоза повышала общее число потребленных калорий и потребление печенья, но существенно не влияла на массу тела, метаболические показатели крови и экспрессию в печени генов, регулирующих транспорт глюкозы (Slc2a2), чувствительность к инсулину (Insr) и процессы аутофагии (Atg8, Becn1, Park2). Поскольку диета кафетерия является наиболее адекватной моделью формирования ожирения у людей, полученные нами результаты ставят под сомнение возможность использования трегалозы для коррекции моделируемого ожирения у людей.

## Introduction

Trehalose (TR) has multiple therapeutic properties, the main of which seem to be the chaperone-like activity and activation of autophagy, which is especially important for neuroprotection (Hosseinpour-Moghaddam et al., 2018; Pupyshev et al., 2022b). Other beneficial TR properties include positive effects on cellular metabolism, carbohydrate and lipid metabolism (Arai et al., 2019; Yaribeygi et al., 2019; Kobayashi et al., 2021), while diabetes, obesity, and neurodegeneration are known to be closely related (Pugazhenthi et al., 2017). Neurodegeneration is considered to be inhibited by TR through the activation of mTOR-independent autophagy (Sarkar, 2013; Tamargo-Gómez, Mariño, 2018).

At the same time, TR significantly affects carbohydrate metabolism, as it can reduce blood glucose levels and insulin resistance (Zhang et al., 2018; Zhang, DeBosch, 2019; Korolenko et al., 2021). TR also has a positive effect on lipid metabolism: it reduces the level of triglycerides in the liver and blood (Stachowicz et al., 2019; Zhang, DeBosch, 2019; Korolenko et al., 2021) and ultimately prevents the development of steatosis dependent on autophagy activity (Zhang et al., 2018; Ren et al., 2019; Su et al., 2025). TR regulates lipid metabolism partly through its effect on the release of adiponectin, which promotes fat burning (Arai et al., 2013; Mizote et al., 2016) through the reduction of the secretion of gastric inhibitory polypeptide GIP, which promotes obesity (Yoshizane et al., 2017) through the effects on the expression of lipoxygenase ALOXE3 and arginase 2, which increase energy consumption (Higgins et al., 2018; Zhang et al., 2019).

In a high-fat diet (HFD)-induced obesity model, TR reduces mesenteric and inguinal fat hypertrophy and brown fat gain (Arai et al., 2019), which is accompanied by an increase in thermogenesis both in C57BL/6 mice and in a genetic model of diabetic obesity, ob/ob mice (Zhang et al., 2018), while in the latter case the result depends on the activity of AMPK, TFEB and UCP1, but not on autophagy (Zhang et al., 2018; Rusmini et al., 2019). In general, the results on the effects of TR on excess body weight are quite contradictory (Arai et al., 2010, 2019; Liu et al., 2013; Sahebkar et al., 2019; Korolenko et al., 2021). Some studies reported a tendency to decrease in the mass of total visceral fat (no more than 5 %) and no significant effect on the body weight in mice kept on HFD after 8-week consumption of 2 % TR (Arai et al., 2013; Liu et al., 2013). According to other data (Korolenko et al., 2021), in db/db mice (a monogenic model of diabetic obesity), threeweek treatment with 2 % TR caused a noticeable decrease in the body weight of mice (more than 10 %) and a general therapeutic effect, in particular, a decrease in the levels of cholesterol, triglycerides, and plasma glucose.

It remains unclear to what extent the fat-reducing effect of TR depends on the type of diet or on mutations that cause obesity. In the present study, we considered that the development of alimentary forms of obesity in mice can be induced both by the consumption of HFD food and by the diet with an increased content of both fats and carbohydrates (carbohydrate-fat diet, “cafeteria diet”, DCaf), which is more common in the human population

In this regard, the study aimed to assess the effect of alimentary consumption of TR on the main metabolic parameters (body weight; food, calorie and water consumption) in C57BL/6 mice with obesity induced by keeping the animals on a carbohydrate-fat diet (DCaf). The biochemical parameters and lipid spectrum of the blood plasma and autophagy, assessed by the expression of autophagy genes in the liver, were also studied.

## Materials and methods

Modeling obesity. All animal manipulations performed during the study met the ethical standards approved by the legal acts of the Russian Federation, the principles of the Basel Declaration and the recommendations of the independent bioethics committee of the Institute of Cytology and Genetics SB RAS (protocol number No. 76 dated 04/07/2021). The experiments were performed in male C57BL/6 mice of the conventional vivarium of the Institute of Cytology and Genetics SB RAS (Novosibirsk, Russia).

Three-month-old animals were kept individually in a cage under a 12-hour light : 12-hour dark regimen, at a temperature of 22–24 °C, and with free access to water and pelleted chow (Assortiment-Agro, Novosibirsk, Russia). After two weeks, the animals were either left on standard diet (n = 22) or transferred to a high-fat, high-carbohydrate diet (DCaf) (n = 19) consisting of unsalted pork lard, biscuits, and standard pelleted chow. DCaf causes obesity in C57BL/6 mice within 18 weeks (Makarova et al., 2013); DCaf content is the closest to the current daily diet in humans and it also allows to assess the consumption of certain dietary components

**Fig. 1.**
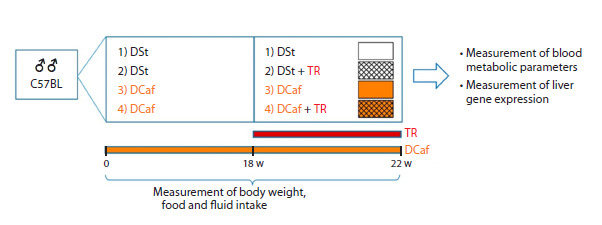
Scheme of the experiment. Abbreviations: DSt – standard laboratory diet, DСaf – cafeteria diet, TR – trehalose

18 weeks after the start of DCaf intake, the animals were divided into four groups (Fig. 1): 1) standard diet (the consumption of standard chow) and water (n = 11); 2) standard diet (the consumption of standard chow) and 3 % TR solution (the rationale behind the TR dosage adopted in the current study was based on our recent study (Pupyshev et al., 2024)) (n = 11); 3) DCaf and water (n = 9); 4) DCaf and 3 % TR solution (n = 10). Throughout the experiment, mice body weight was measured weekly while food intake was measured three times a week.

Blood biochemistry. After four weeks of trehalose consumption, the animals were sacrificed, blood and liver samples were collected. Blood serum samples were collected as described previously (Goncharova et al., 2016). Metabolic parameters (ALT, creatinine, glucose, triglycerides, cholesterol, and high-density lipoproteins (HDL)) were determined in the blood serum using an AU 680 Biochemical Analyzer (Beckman Coulter, USA).

Analysis of gene expression. Real-time qPCR was used to evaluate the relative expression levels of liver genes involved in the regulation of carbohydrate metabolism (Insr, encoding the insulin receptor; Slc2a2, encoding the glucose transporter type 2 GLUT2) and autophagy activity (Atg8, encoding the autophagy protein LC3-II; Becn1, encoding the protein Beclin 1; Park2, encoding the protein Parkin), as well as the reference genes Hprt1, B2m, Ppia

Total RNA was isolated from liver samples using the ExtractRNA kit (Evrogen, Moscow, Russia) according to the manufacturer’s instructions. cDNA synthesis was performed using the MMLV RT kit (Evrogen) according to the manufacturer’s protocol (https://evrogen.ru/products/cdna/synthesis/mmlv). The resulting cDNA samples were analyzed by qPCR on a LightCycler-480 II thermal cycler (Roche, Switzerland) using the BioMaster HS-qPCR SYBR Blue (2×) reagent kit (Biolabmiks, Novosibirsk, Russia), with forward (F) and reverse (R) primers (150 nM each) for the studied genes. Primers used for the target genes: Atg8 [F: 5′-AAA GAG TGG AAG ATG TCC GGC-3′ and R: 5′-ACC AGG AAC TTG GTC TTG TCC-3′], Becn1 [F: 5′-GAA CTC ACA GCT CCA TTA CTT A-3′ and R: 5′-ATC TTC GAG AGA CAC CAT CC-3′], Insr [F: 5′-ATC CTC GAA GGT GAG AAG AC-3′ and R: 5′-TGA TAC CAG AGC ATA GGA GC-3′], Park2 [F: 5′-GGT CCA GTT AAA CCC ACC TAC-3′ and R: 5′-TTA AGA CAT CGT CCC AGC AAG-3′], Slc2a2 [F: 5′-GGCTAATTTCAGGACTGGTT-3′ and R: 5′-TTTCTTTGCCCTGACTTCCT-3′]. Primers used for the reference genes: B2m [F: 5′-GTC TTT CTA TAT CCT GGC TCA-3′ and R: 5′-ATG CTT GAT CAC ATG TCT CG- 3′], Hprt1 [F: 5′-TAC CTA ATC ATT ATG CCG AGG A-3′ and R: 5′-GGT CAG CAA AGA ACT TAT AGC C-3′], Ppia [F: 5′- AAA GTT CCA AAG ACA GCA GAA AA-3′ and R: 5′-GCC AGG ACC TGT ATG CTT TAG-3′]. The relative concentration of the tested cDNA was determined using Light- Cycler 480 software (1.5.1 version) and calibration curves.

Statistics. The data were analyzed using STATISTICA 10.0 (StatSoft, TIBCO Software Inc., Palo Alto, CA, USA). The results were expressed as the mean ± standard error of the mean. A two-tailed Student’s t-test was used to compare sample means. A statistically significant level of differences was accepted at p < 0.05 (two-tailed).

## Results

**Modeling diabetic obesity**

**Fig. 2.**
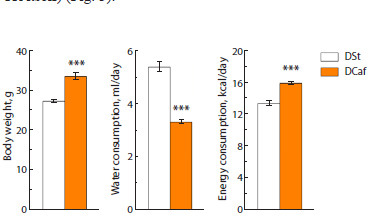
Body weight, water consumption, and total energy intake of mice maintained on standard diet (n = 22) or cafeteria diet (n = 19) for 18 weeks before they were given 3 % trehalose solution. *** p < 0.001 vs. DSt.

Before the start of treatment with 3 % TR solution (after 18 weeks of maintenance on DSt or DCaf), mice from comparison groups 1 and 2, as well as obese animals from groups 3 and 4, did not differ in body weight, food consumption, or taste preferences (see the Table, Fig. 2).

**Table 1.**
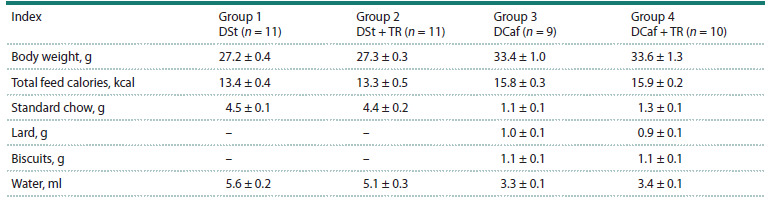
Body weight, water consumption, total energy and dietary component intake in mice in four groups Note. Mice in groups 1 and 2 maintained on standard diet (DSt) and in groups 3 and 4 maintained on the cafeteria diet (DCaf ) for 18 weeks before the start of treatment with 3 % trehalose (TR) solution in groups 2 and 4. Daily component consumption is indicated. Results are expressed as M ± m (n, number of animals).

Mice that consumed DCaf for 18 weeks developed excess body weight (obesity): body weight increased by 25 % (p < 0.001), energy consumption increased by 20 % (p < 0.001), and water consumption, on the contrary, was reduced by 40 % (p < 0.001) compared to mice in the control group (Fig. 2).

In animals kept on DCaf, the indicator of hyperglycemia (blood glucose level) increased by 35 % (see below), and this differs from the effect of DCaf in other studies, where blood glucose level increased more significantly (Parafati et al., 2015), or in genetic models of obesity, ob/ob and db/db mice (Pelletier et al., 2020; Korolenko et al., 2021). In our experiment, excess weight increase was not accompanied by the development of high hyperglycemia.

**Effects of TR on metabolic parameters in mice kept on standard laboratory diet**

**Fig. 3.**
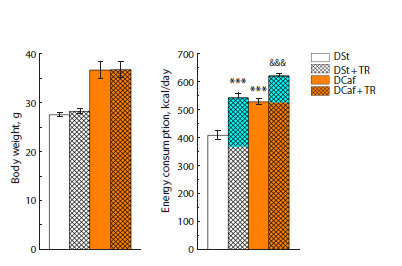
Body weight and total energy intake in mice maintained on standard diet and cafeteria diet for four weeks during which the animals consumed water or 3 % trehalose solution. The blue color shows the number of kcal obtained with drinking 3 % TR solution. *** p < 0.001 vs. DSt; &&& p < 0.001 vs. DCaf and water group (t-test).

**Fig. 4.**
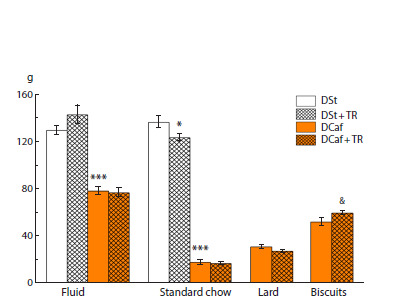
Total fluid and various food components intake of mice maintained on standard diet or cafeteria diet for four weeks during which the animals consumed water or 3 % trehalose solution. * p < 0.05, *** p < 0.001 vs. DSt; & p < 0.05 vs. DCaf and water group (t-test).

Consumption of a 3 % TR solution for four weeks in control mice did not affect the body weight of the animals (Fig. 3). These mice ate less food (p < 0.05), while they showed a tendency for an increase in fluid consumption by 10 % (Fig. 4). Taking into account the caloric supplementation from TR in drinking, the total kcal consumption in control mice drinking a 3 % TR solution was more than 30 % higher than in control animals drinking water (p < 0.001) (Fig. 3).

**Effects of TR on metabolic parameters in mice kept on cafeteria diet**

Despite our expectations, the body weight of mice kept on DCaf did not change under the influence of TR (Fig. 3). TR also had a weak effect on their consumption of standard food, liquid, or lard but increased the consumption of the carbohydrate component, biscuits (Fig. 4). TR significantly increased the number of calories consumed, by 18 % (taking into account the unchanged body weight in these animals compared to the group drinking water and the caloric supplementation from TR itself) (Fig. 3).

**Fig. 5.**
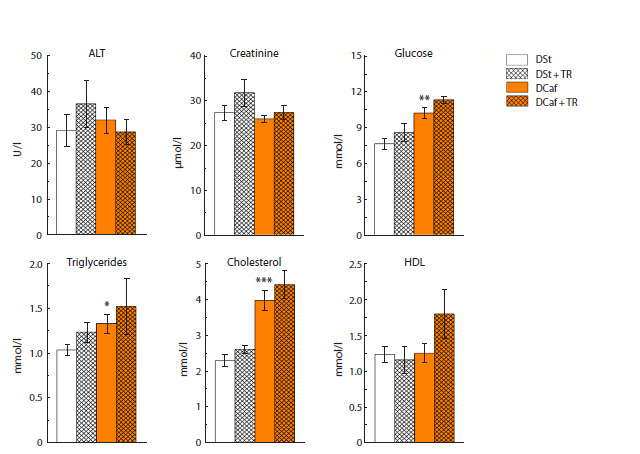
Blood biochemical parameters in mice maintained on standard diet or cafeteria diet and given water or 3 % trehalose solution for four weeks. * p <0.05, ** p < 0.01, *** p < 0.001 vs. DSt.

TR did not produce significant changes in metabolic blood indices in mice maintained on DCaf or in the control group (except for a noticeable trend (p < 0.07) in blood glucose growth) (Fig. 5). The maintenance of mice on DCaf per se influenced the overall metabolism to a certain extent increasing the levels of glucose (p < 0.01), triglycerides (p < 0.05), and especially those of blood cholesterol (p < 0.001), i. e. changes in carbohydrate and lipid metabolism were registered.

**Effects of TR on transcription of carbohydrate metabolism and autophagy genes in mice kept on cafeteria diet**

**Fig. 6.**
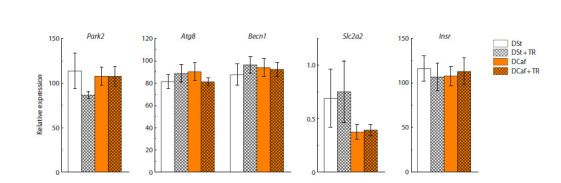
The mRNA levels of autophagy (Park2, Atg8, Becn1) and carbohydrate metabolism (Slc2a2, Insr) genes in the liver of mice kept on standard diet or cafeteria diet and given water or 3 % trehalose solution for four weeks

Neither DCaf nor TR consumption affected the expression of genes regulating glucose uptake from blood (Slc2a2, Insr) or related to autophagy activity (Atg8, Becn1, Park2) in the liver of mice (Fig. 6).

## Discussion

The maintenance of mice on DCaf for 18 weeks was accompanied by an increase in caloric intake (by 20 %) and, as a result, the development of excess body weight (by 25 %). That allowed us to study a normalizing effect of TR on body weight gain found in previous studies in db/db mice (Korolenko et al., 2021). TR as an energy substrate (Sato et al., 1999) increased caloric energy intake both in mice of the control group and in mice given DCaf. Notably, in both groups of mice, an increase in caloric intake mainly associated with TR intake did not augment body mass (Fig. 3). Hence, TR stimulates caloric energy expenditure without affecting body weight. To some extent, this is in line with previous findings that demonstrated the ability of long-term TR treatment to stimulate caloric expenditure in thermogenesis and brown fat burning processes in C57BL/6 mice kept on both standard and high-fat diets (Arai et al., 2013, 2019) and in obese ob/ob mice (Zhang et al., 2018).

Another possible way of regulation by alimentary TR involves the induction of cellular starvation accompanied by activation of cytoprotective autophagy (DeBosch et al., 2016; Mayer et al., 2016; Zhang, DeBosch, 2019). The mechanism is associated with inhibition of the GLUT8 glucose transmembrane transporter, thereby causing energy deficiency in liver cells leading to activation of the adenosine monophosphatedependent kinase AMPK and its pleiotropic metabolic effect including inhibition of biosynthesis, energy consumption, and activation of autophagy. In the present study, TR appeared to lose its ability to simulate the fasting effect (Zhang, DeBosch, 2019), which leads to autophagy activation. The attenuation of the regulatory properties of TR here is consistent with its lack of effect on the transcription of autophagy genes responding to TR by elevation in our other studies (Pupyshev et al., 2022a). The reason for the weakening of the regulatory properties of TR in C57BL/6 mice remains unclear. Such a switch of the effect of TR does not seem to depend on the diet, as the effects of TR were similar both in control mice and in DCaf-given ones. Perhaps, in the conditions in our study (3 % trehalose, 28 days), there is an escape of TR from the quantitative energy cleavage described earlier (Sato et al., 1999), and then the contradiction between the growth of calories consumed and the lack of weight gain is smoothed out.

The loss of regulatory properties of TR in mice in our experiment is not consistent with the effects of TR revealed in db/db mice with diabetic obesity (Korolenko et al., 2021). In db/db mice, TR reduced the body mass (by more than 10 %),reduced blood glucose levels, and produced a general recovery effect by lowering blood cholesterol and triglycerides. However, in studies in rabbits, as here, a weak effect of TR on the blood lipid spectrum was found (Sahebkar et al., 2019).

Given the similarity of the model of obesity in DCaf-given mice with the typical development of obesity in humans, the results question the use of TR to correct this most common type of obesity in humans. At the same time, our results do not exclude the approach of treating patients with severe obesity with TR because such treatment was successful for mice with almost 50 % excess weight (Korolenko et al., 2021).

## Conclusion

Based on the well-known effect of TR on simulation of starvation (induction of autophagy) and reduction of excess body weight in db/db mice, a study of its effect in C57BL/6 mice given carbohydrate-fat diet (DCaf), a model an alimentary obesity in humans, was performed. In the control and obese (by 25 %) mice, TR (3 % solution drinking, 28 days) augmented significantly the number of calories consumed while this increase in energy consumption was not accompanied by an increase in body weight gain of the mice. The excess in calories consumed might be spent on enhancing the processes of thermogenesis and brown fat burning (Arai et al., 2013, 2019). Trehalose produced only a tendency of increase in blood metabolic parameters (glucose, cholesterol, triglycerides, HDL) and had no effect on the expression of genes regulating carbohydrate metabolism (Slc2a2, Insr) or autophagy genes (Atg8, Becn1, Park2). In the present study, in mice kept on DCaf, TR did not demonstrate an ability to reduce diabetes and obesity induced by DCaf, nor useful properties for the correction of common dietary type of obesity in humans.

## Conflict of interest

The authors declare no conflict of interest.
